# Cross-Sectional Study on Influenza Vaccination, Germany, 1999–2000

**DOI:** 10.3201/eid0812.010497

**Published:** 2002-12

**Authors:** Sybille Rehmet, Andrea Ammon, Günter Pfaff, Nikolaus Bocter, Lyle R. Petersen

**Affiliations:** * Robert Koch-Institut, Berlin, Germany; † Gesellschaft für Technische Zusammenarbeit (GTZ), Eschborn, Germany; ‡ Landesgesundheitsamt Baden-Württemberg, Stuttgart, Germany; § Akademie für Öffentliches Gesundheitswesen, Düsseldorf, Germany; ¶ Centers for Disease Control and Prevention, Fort Collins, Colorado, USA

**Keywords:** Influenza, vaccination, Germany, epidemiology

## Abstract

To assess influenza vaccination coverage in Germany, we conducted a nationwide telephone survey in November 1999 in adults (>18 yrs) using random-digit dialing. Overall, 23% of 1,190 survey participants reported having been vaccinated (adjusted 18%) with 16% (adjusted 15%) in former West Germany versus 35% (adjusted 32%) in former East Germany. Immunization rates for vaccination target groups were lower in West Germany (21%) than in East Germany (40%). Seven percent of health-care workers were immunized. Previous influenza vaccination, positive attitudes towards immunization, and having a family physician increased the rate of vaccination; fear of adverse effects lowered the rate. Family physicians performed 93% of the vaccinations, which suggests their key role in improving low vaccination coverage in Germany. The fact that >71% (850/1,190) of participants belonged to at least one of the vaccination target groups recommended by the German Standing Commission on Immunization emphasizes the need to focus the definition of target groups.

Ten years after the reunification of the former East and West Germany, the Federal Republic of Germany still shows the effects resulting from combining two different health-care systems after being apart for 50 years. Even though the health-care systems were merged soon after reunification, differences in health-care practices persist, especially in regard to preventive medicine and immunization, which had a much higher priority in the former East Germany. For example, a pilot study undertaken in Berlin, Stuttgart, and Chemnitz during the influenza season 1998–1999 showed much higher influenza vaccination rates in East Berlin and Chemnitz in the former East Germany (called former East in this paper) than in West Berlin and Stuttgart in the former West Germany (called former West in this paper) (1,2).

In general, population-based studies of influenza vaccination coverage for a country do not exist. A Canadian study found 13.8% influenza vaccination coverage in fall and winter 1990–1991 (3). Most studies on influenza vaccination coverage investigate specific groups such as the elderly (4–13), patients from general practices (14,15), or hospitalized patients (16).

This lack of nationwide, population-based studies, along with the findings of the pilot study showing markedly different vaccination rates in several German cities, prompted the nationwide, population-based survey reported here. The goals of our survey were to determine the influenza immunization rates in areas of the former East and West during the 1999–2000 influenza season, the proportion of the German population included in specific vaccination target groups recommended by the German Standing Commission on Immunization, the vaccination rates among these target groups, and factors that might influence immunization rates in areas of the former East and West.

## Methods

### Background

Germany has a population of 82 million; 14 million live in areas of the former East and 68 million in areas of the former West (17). The German Standing Commission on Immunization has recommended that the following groups receive influenza vaccination: 1) persons >60 years old, 2) persons with chronic illness, 3) health-care professionals, and 4) persons who have extensive contact with the general public. The first three groups comprise an estimated 35% of the general population and 42% of the adult population of Germany (Arbeitsgruppe Seuchenschutz, Robert Koch-Institut, 7 September 1999). The size of the fourth group is unclear because of its widely applicable definition. For the target groups, the influenza vaccination period started in September 1999. Vaccinations were administered free of charge.

### Survey

The target survey population included noninstitutionalized persons >18 years of age living in Germany. A standardized, pretested questionnaire was administered by telephone on November 8 and November 22, 1999. Sample households were chosen by random-digit dialing by using a computer-generated list of possible telephone numbers. Approximately half of the telephone numbers on the list had prefixes in the former East. However, the proportion of working phone numbers was lower in the former East, so the actual number of former East residents who answered the phone and agreed to participate was <50% of all participants. The person who initially answered the telephone was eligible to be interviewed; to be eligible, persons also had to be >18 years of age, live in a private household, and have sufficient knowledge of German to be able to understand and answer the questions. If persons <18 years of age answered the phone, they were asked if an adult was present in the household, and an attempt was made to interview that person. After verbal, informed consent was obtained from the participant, we administered a questionnaire that gathered information about demographics, individual risk factors for contracting influenza, history of vaccination, general attitude towards immunization, perceived efficacy and adverse effects of the influenza vaccine, as well as other factors that might influence whether a person was likely to have been vaccinated.

Participants were counted as being vaccinated in the current influenza season if they reported having received an influenza vaccination after September 1, 1999. Persons were counted as being in a target group recommended by the German Standing Commission on Immunization if they reported one or more of the following: 1) age of >60 years, 2) chronic illness currently requiring regular medical supervision or treatment, 3) work in a health-care environment, in which at least half the working day involved interacting with patients, and 4) working at a job in which more than half the working day was spent with people who were not their colleagues. To provide added specificity, these latter two groups were narrower in scope than those defined by the Standing Commission on Immunization. In the following text, these two groups are summarized as “professional exposure.” Immunization rates were adjusted for age, sex, and residence in areas of the former East and West according to the official 1998 population data (17).

## Results

### Study Population

Dialing 4,863 numbers yielded 2,057 actual connections. Of these, 25 were discarded because the person who answered the phone had insufficient knowledge of German and 24 because no present household member was >18 years of age. Of the remaining 2,008 persons, 1,190 (59%) participated in the survey. Of the participants, 718 (60%) reported living in the former West, 462 (39%) in the former East, and 10 (1%) did not report location of residence. Sixty-three percent (452) of survey participants living in the former West and 60% (276) living in the former East were women. The median age was 47 years for persons living in the former West and 51 years for those in the former East. Three percent (33) of survey participants were not German citizens.

### Immunization Status

Of the 1,183 participants who reported their vaccination status, 277 (23%) reported having been vaccinated since September 1. Reported immunization rates were much lower in the former West Germany (16%; 115/715) than in the former East (35%; 159/459). The study population differed from the general German population with regard to age structure, gender, and place of residence in the former East or West as reported in the official population data from 1998 (17). The estimated immunization rate for the whole country, adjusted for age, gender, and place of residence (former West or East), was 18% (95% confidence interval [CI] 16% to 21%). The age- and gender-adjusted immunization rate in the former West was 15% (95% CI 13% to 18%), a figure significantly lower than 32% (95% CI 28% to 37%) in the former East.

Sixty-eight percent (489) and 78% (361) of survey respondents living in the former West and East, respectively, reported at least one characteristic that placed them in an influenza immunization target group. Among target group members, reported vaccination rates were nearly twice as high among those living in areas of the former East (40%; 142/358) than those living in areas of the former West (21%; 101/486). Immunization rates were higher among all target subgroups in areas of the former East (Table 1). Vaccination rates were particularly low among health-care workers (7% [95% CI 1% to 13%] in the former West and 10% [95% CI 1% to 19%] in the former East).

**Table 1:** Immunization rates in the target groups for influenza vaccination, former West and East Germany, November 1999

Target groups	Former West Germany			Former East Germany		
	Vaccinated	% (95% CIa)	Total	Vaccinated	% (95% CIa)	Total
>60 yrs of age	78	37 (30% to 43%)	213	90	55 (47% to 63%)	163
Chronic illness	51	31 (24% to 39%)	164	81	49 (41% to 57%)	165
Professional exposure	21	9 (5% to 13%)	243	39	25 (19% to 33%)	155
Health-care workers	4	7 (2% to 16%)	60	2	10 (1% to 30%)	21
Workers with public contact	17	9 (6% to 14%)	183	37	28 (20% to 36%)	134
One or more of the above	101	21 (17% to 25%)	486 b	142	40 (35% to 45%)	358b

aCI, confidence interval. bTotals do not include three persons from former West Germany and three persons from former East Germany for whom information on immunization status was not available.

### Possible Factors Influencing Immunization

We restricted our analyses of factors influencing the immunization rate to those reported by the 587 participants in the target subgroups (aged >60 years, chronically ill, and working in the health-care profession) and for whom information on area of residence in former East or West, as well as vaccination status, was available. Among these persons, those reporting a positive overall attitude towards immunization, those believing that the vaccine is efficacious, and those having had received an influenza vaccination in previous years were much more likely to have been vaccinated in the current immunization period (Table 2). Those reporting fear of contracting influenza from vaccination and fear of other adverse effects had lower immunization rates.

**Table 2:** Factors significantly influencing likelihood of an influenza vaccination during the immunization period (1999–2000) for 587 survey participants, by area of residence, November 1999a–c

		Former West Germany				Former East Germany			
		Vaccination		OR	95% CI	Vaccination		OR	95% CI
Yes	No	Yes	No
Influenza vaccination in previous years	Yes	69	52	10.4	5.8% to 19.1%	101	42	18.4	8.9% to 40.9%
	No	24	190			12	93		
Positive attitude towards immunization in general	Yes	78	164	7.8	1.9% to 68.9%	108	103	6.3	1.3% to 58.8%
	No	2	33			2	12		
Belief in efficacy of vaccine	Yes	84	159	9.7	2.4% to 85.3%	111	94	12.3	2.9% to 110.9%
	No	2	34			2	21		
Belief that influenza is a severe disease	Yes	73	200	3.1	0.7% to 28.3%	90	106	2.3	0.7% to 10.4%
	No	2	17			4	11		
Information from the media	Yes	48	134	0.9	0.5% to 1.4%	80	77	1.8	1.0% to 3.2%
	No	44	105			32	56		
Regular family physician	Yes	92	217	11.8	1.9% to 490.6%	112	123	5.0	1.1% to 47.2%
	No	1	28			2	11		
Consultation with physician since September 1, 1999	Yes	81	152	4.4	2.2% to 9.6%	109	87	14.6	5.1% to 57.9%
	No	11	91			4	47		
Vaccination offer during consultationd	Yes	63	31	13.5	6.7% to 27.9%	83	35	4.7	2.5% to 9.2%
	No	18	121			26	52		
Fear of contracting influenza through vaccination	Yes	17	111	0.2	0.1% to 0.5%	29	62	0.5	0.3% to 0.8%
	No	55	84			60	59		
Fear of adverse effects	Yes	5	54	0.1	0.0% to 0.3%	11	24	0.3	0.1% to 0.7%
	No	80	87			94	62		

aOR, odds ratio; CI, confidence interval. bDenominator varies because persons who indicated “don’t know” were not included in the analysis. cTarget groups included those >60 years of age, the chronically ill, and those who worked as professionals in the health-care sector.  dOnly persons having seen a physician since September 1, 1999.

Participants who had read at least one media article in the fall about influenza immunization or who thought influenza a serious illness had similar or slightly higher immunization rates than those without these characteristics (Table 2). Although all the participants included in this analysis reported at least one characteristic of the target subgroups, only 21% (121/587) considered themselves to be at increased risk of contracting influenza compared with the general public.

### Role of the Family Physician

Vaccinated survey participants in the key target vaccination groups (those aged >60 years, those with chronic illness, and health-care professionals) reported that family physicians performed 93% (84/90) and 94% (106/113) of vaccinations in the former West and East, respectively. Among those participants not vaccinated, more than half (52% in the former West and 63% in the former East) stated that they would have agreed to be vaccinated on the advice of a physician. Persons who reported having a regular family physician had higher immunization rates (Table 2). Those who had visited a physician since September 1, 1999, were more likely to have been immunized than those who had not. During the visit, if the physician had advised immunization, the probability of being immunized increased further. However, only 40% (94/233) and 60% (118/196) of those living in the former West and East, respectively, who reported having had a consultation since September 1, 1999, also reported having been offered influenza vaccination by their physician.

### Immunization in the Workplace

Of the working survey participants, 18% (70/386) and 15% (33/228) in the former West and East, respectively, indicated that influenza immunization had been offered at the workplace. For those employed in health-care professions, these percentages were 35% (21/60) and 43% (9/21) in the former West and East, respectively. Five (6%) of the 80 immunized working participants were immunized at the workplace.

## Discussion

We estimate that, as of November 22, 1999, 18% of the German population >18 years of age had received influenza vaccination for the 1999–2000 influenza season. This percentage corresponds to the 20% maximum estimate of the immunization rate calculated from the number of vaccine doses sold for the immunization period 1999–2000, assuming all doses sold were given (13.1 million doses for the 1999 influenza vaccination period; data provided by the suppliers). The estimated immunization rate of 18% is substantially lower than the target of 42%, based on percentage of the adult population comprising the key target groups for vaccination. Vaccination rates were nearly twice as high among persons living in the former East than in the former West, despite the fact that the two health-care systems have been unified for almost 10 years. Similar geographic differences in rates existed among all recommended vaccination target groups. Nevertheless, vaccination rates were inadequate among the key target groups of the elderly and those chronically ill in all areas; only approximately one third of persons in the former West and one half in the former East were vaccinated. Another finding was the low vaccination rates among surveyed health-care workers; however, few health-care workers were surveyed.

In Germany, the attitudes and practices of the family physicians may be a critical factor in influencing influenza vaccination rates. Our study showed that persons in key target groups for vaccination (age >60 years, chronic illness, health-care professionals) who had had a regular family physician and had had a recent medical consultation during which the physician offered vaccination were much more likely to have been vaccinated. These results are consistent with other studies showing the importance of physicians or health-care personnel in motivating people for influenza vaccination ( 3,9). Almost all vaccinations, both in the former West and East, were given by a family physician, and over half the nonimmunized participants stated they would have agreed to be vaccinated on advice of a physician. Family physicians thus have a substantial opportunity to improve immunization coverage by more actively and frequently recommending vaccination, especially to persons belonging to a risk group. The fact that 60% of participants belonging to key target groups for influenza vaccination in the former West and 40% in the former East who had seen their physician during the immunization period were not actively offered vaccination indicates many missed opportunities for vaccination. A study by Booth et al. (14) shows that 71% to 82% of general practitioners reported having routinely offered influenza vaccination to patients from risk groups. Although similar data do not exist for Germany, that only 40% to 60% of our survey participants reported having been offered immunization suggests that fewer general practices routinely offer vaccination. Perenboom et al. (18) found that, in the Netherlands, when general practitioners invited their chronically ill patients to be vaccinated, vaccination coverage increased among this group to 75.5%, compared with 42% for the same risk group found in the National Health Interview Survey.

Health-care workers represent a specific target group with an extremely low vaccination rate. Because the number of health-care workers who participated in this study was low (81 workers), results must be carefully interpreted. Our results, however, were confirmed by later studies (Hallauer et al., unpub. data; 6). Health-care workers might not be reached by family physicians, but rather by alternative interventions such as vaccination programs at the workplace. However, this group needs further investigation regarding targeted interventions to increase vaccination coverage.

Among those in key target groups for vaccination, persons who had received an influenza immunization in a previous year were much more likely to have been immunized during the current period. This conclusion is consistent with the findings of several previous studies (19–28). Therefore, a concerted effort to increase vaccination coverage in target groups in 1 year might have a positive impact on revaccination in the following years. This success rate might be one reason for the persistently higher vaccination rates among persons living in the former East.

Our results suggest several possibilities for improving influenza vaccination rates in Germany. One possibility would be to better focus the target populations for influenza vaccination. We found that approximately 70% of the population fit into a target vaccination group, largely due to the category comprising public exposure in the workplace. Despite the fact that our definition for this target group (persons who spend more than half their working day dealing with many people not their colleagues) was narrower than that used by the German Standing Commission on Immunization (public exposure in the workplace), this group included almost half the participants belonging to target groups. Were this group defined more precisely, the criteria could be communicated more clearly to family physicians and employers and thus make the indications for immunization less ambiguous.

Fear of contracting influenza through immunization and fear of adverse effects had a negative impact on the immunization rate, as seen in other studies (3,9,19,21,22,24,26,28,29–33). Health information messages, particularly those given by physicians aimed at reducing these fears, may have a beneficial effect on vaccination rates. Earlier research suggests that the self-perception of influenza risk is often inaccurate (1,2). Our study confirms this finding; only about one fifth of those participants belonging to a target group were, in their own opinion, at higher risk of becoming more severely ill from influenza than the general population. Again, we suggest that targeted information about risk factors for influenza and complications should be enforced. Another way to increase vaccination rates may be to improve workplace immunization programs, particularly for health-care workers. We found that <40% of health-care workers interviewed in this study reported having a workplace influenza immunization program. With <10% of the health-care workers reporting having been vaccinated, the existing programs must be largely ineffective.

Our study has several limitations. The study population differed from the general population in age, gender, and place of residence. To avoid possible biases because of these differences, we used standardized figures. Influenza vaccination was self-reported; because the survey was anonymous, confirmation of vaccination status was not possible. In addition, because we were unable to repeatedly call households on different days if nobody answered the phone on the first try, persons who spent more time at home were probably more likely to participate, resulting in an overrepresentation of persons in certain vaccination target groups (age >60 years, chronic illness). Persons who lived in households without telephones or could not speak German were also not sampled. If these groups have a lower vaccination rate, our estimated vaccination rate will then have been overestimated.

The timing of the survey (late November) may have led to an underestimate of the true vaccination rate because participants might have been vaccinated later in the season. No studies from Germany on vaccine uptake during the vaccination period have been available up to now. However, the overall vaccination rate shows that the study (18%) and the vaccination rate calculated by using the number of doses sold (20%) correspond closely and suggests that the number of persons vaccinated in the later months of the immunization period was low. Although the German influenza vaccination experience reported in this study suggests areas for improvement, the circumstances resulting from the German reunification also demonstrate the long-term benefits of a sustained, concerted effort to improve influenza vaccination rates.
